# Nux—Cerebro-Spinal Meningitis

**Published:** 1881-05

**Authors:** E. Bayard

**Affiliations:** New York City


### CEREBRO-SPINAL MENINGITIS IN A HORSE, CURED BY A C.M. POTENCY.

BY E. BAYARD, M.D., NEW YORK CITY.

Owing to the sudden and sharp changes during this severe winter, many horses in this city, from hard work and rapid driving in the continuous sleighing of the season, were attacked with an acute form of cerebro-spinal meningitis, and died of the disease. The attack commenced with general dullness, loss of appetite, stiffness, and difficulty of moving the hind-quarters, and if not at once arrested, it overwhelms the animal.

A friend, owning a valuable horse, told me the animal was dull, had lost his appetite, could not move about the stall, had apparent stiffness of the hind-quarters, and asked if homœopathy could give him relief. Knowing that *Nux vomica* produced rigidity, torpor, heaviness and paralysis of the limbs, particularly rigidity and tension of the hams with insensibility of the legs, I said I thought it would and I gave him *Nux vomica* 100 M. (Swan), three doses, to be taken every two hours. Twenty-four hours after, the gentleman informed me that his horse was eating his food with relish; that all stiffness in the hind-quarters had left him, and he was apparently well.

I think this a fair expression of a scientific precision of the homœopathic law, and shows at the same time the keen susceptibilities of the horse to a similar irritant.

